# Potential Role of VHL, PTEN, and BAP1 Mutations in Renal Tumors

**DOI:** 10.3390/jcm12134538

**Published:** 2023-07-07

**Authors:** Krisztián Szegedi, Zsuzsanna Szabó, Judit Kállai, József Király, Erzsébet Szabó, Zsuzsanna Bereczky, Éva Juhász, Balázs Dezső, Csaba Szász, Barbara Zsebik, Tibor Flaskó, Gábor Halmos

**Affiliations:** 1 Department of Urology, Faculty of Medicine, University of Debrecen, 4032 Debrecen, Hungary; szegedi.krisztian@med.unideb.hu (K.S.); flash@dote.hu (T.F.); 2 Doctoral School of Pharmaceutical Sciences, University of Debrecen, 4032 Debrecen, Hungary; 3 Department of Biopharmacy, Faculty of Pharmacy, University of Debrecen, 4032 Debrecen, Hungary; szabo.zsuzsanna@pharm.unideb.hu (Z.S.); kiraly.jozsef@pharm.unideb.hu (J.K.); erzsebet.szabo@med.unideb.hu (E.S.); barbara.zsebik@pharm.unideb.hu (B.Z.); 4 Division of Clinical Laboratory Science, Department of Laboratory Medicine, Faculty of Medicine, University of Debrecen, 4032 Debrecen, Hungary; kallai.judit@med.unideb.hu (J.K.); zsbereczky@med.unideb.hu (Z.B.); 5 Department of Pediatrics, Faculty of Medicine, University of Debrecen, 4032 Debrecen, Hungary; juhasze@med.unideb.hu; 6 Department of Pathology, Faculty of Medicine, University of Debrecen, 4032 Debrecen, Hungary; bdezso@med.unideb.hu (B.D.); szasz.csaba@med.unideb.hu (C.S.); 7 Department of Oral Pathology and Microbiology, Faculty of Dentistry, University of Debrecen, 4032 Debrecen, Hungary

**Keywords:** renal tumor, renal cell carcinoma (RCC), angiomyolipoma (AML), mutation, genetic polymorphism, VHL, PTEN, BAP1

## Abstract

The genetic profiling of renal tumors has revealed genomic regions commonly affected by structural changes and a general genetic heterogeneity. The VHL, PTEN, and BAP1 genes are often mutated in renal tumors. The frequency and clinical relevance of these mutations in renal tumors are still being researched. In our study, we investigated VHL, PTEN, and BAP1 genes and the sequencing of 24 samples of patients with renal tumors, revealing that VHL was mutated at a noticeable frequency (25%). Six of the investigated samples showed mutations, and one genetic polymorphism (rs779805) was detected in both heterozygote and homozygote forms. PTEN gene mutation was observed in only one sample, and one specimen showed genetic polymorphism. In the case of the BAP1 gene, all of the samples were wild types. Interestingly, VHL mutation was detected in two female patients diagnosed with AML and in one with oncocytoma. We assume that VHL or PTEN mutations may contribute to the development of human renal cancer. However, the overall mutation rate was low in all specimens investigated, and the development and prognosis of the disease were not exclusively associated with these types of genetic alterations.

## 1. Introduction

Renal cell carcinoma (RCC) is an aggressive disease that represents 2–3% of all adult cancers and is the 10th most common cancer worldwide. About 20% to 30% of patients have distant metastases at diagnosis [1,2,3,4]. Moreover, it is the third most common urological cancer after prostate and bladder cancers; however, it has the highest mortality rate, at more than 40% [1,3,4]. Among all types, clear cell renal cell carcinoma (ccRCC) is the most common subtype, which accounts for 75–80% of all RCC cases [1,2,3,4]. If detected early, localized RCC can be treated surgically, and a 5-year survival rate approaching 85% can be achieved for patients [1]. Unfortunately, about 40–50% of patients relapse after nephrectomy or present at diagnosis with metastatic disease (mRCC) [1,2]. Despite the therapeutic improvements made in the past decade, mRCC is still an incurable disease. Apart from surgery, it is resistant to both chemotherapy and radiotherapy [1]. 

Other studies have also found that a significant proportion of solid renal masses are histologically benign [5]. For example, renal angiomyolipoma (AML) and oncocytoma (oxyphilic adenoma) are benign types of renal cell neoplasms and affect a significant proportion of patients who undergo surgery [5,6].

Therefore, it is necessary to improve our understanding of the genetic background of RCC’s pathogenesis and to develop novel targeted therapies by improving approaches of personalized therapies based on the individual genetic features of patients [1,2].

During the exploration of the molecular biology of ccRCC both at a genetic and epigenetic level, a complex interplay was observed which leads to the generation of an altered proteome profile [7,8,9,10,11,12]. The central genetic event in the pathogenesis of ccRCC is the biallelic inactivation of the Von Hippel–Lindau (VHL) gene, either due to somatic mutations or promoter hypermethylation [7,8,9,10]. Apart from VHL gene inactivation, other epigenetic genes also play a contributing role in ccRCC development, which mainly includes phosphatase and tensin homolog (PTEN) and breast cancer type-1 (BRCA-1)-associated protein-1 (BAP1) [1,4,11]. 

**Figure 1:**
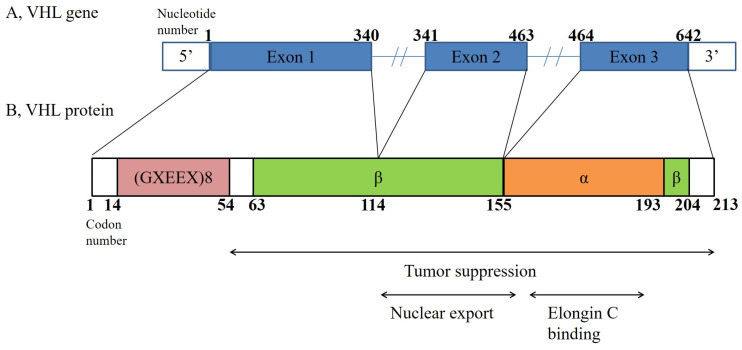
VHL gene structure (**A**) and VHL protein structure (**B**). The gene spans 10 kb and comprises three exons. The VHL protein is produced in two forms: an 18 kDa protein and a 30 kDa protein. The protein encoded by this gene is a component of the protein complex that includes elongin B, elongin C, and cullin-2, and it possesses ubiquitin ligase E3 activity. This activity is thought to be the main action of the VHL protein. The VHL protein consists of 214 amino acids and has two structural domains: the α-domain and the β-domain [1,6].

Von Hippel–Lindau (VHL) disease (OMIM 193300) is an autosomal dominant hereditary cancer syndrome that predisposes an individual to the development of cerebellar hemangioblastomas, retinal angiomas, clear cell renal cell carcinomas, neuroendocrine tumors, and pheochromocytomas. Von Hippel–Lindau disease is caused by germline mutations in the VHL tumor suppressor gene, which is located on the short arm of chromosome 3 (3p25-26) (Figure 1). 

Mutation of the VHL gene causes a protein’s normal function to be lost or altered [12,13,14]. Up to 90% of sporadic ccRCC patients harbor VHL gene inactivation; however, mutations are associated with only 50% of these cases. Moreover, 10–20% of the patients exhibited promoter hypermethylation [9,13,15]. 

**Figure 2:**
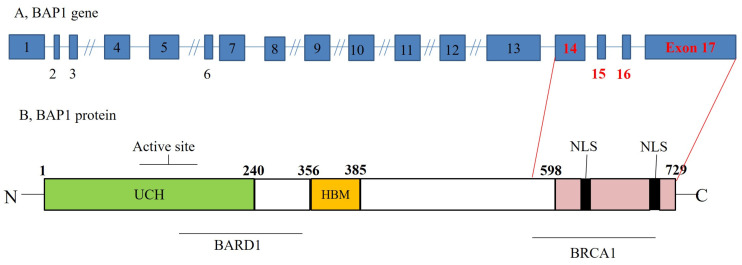
BAP1 gene structure (**A**) and BAP1 protein structure (**B**). In humans, BRCA1-associated protein-1 (BAP1) is encoded by the BAP1 gene located on the short arm of chromosome 3 (3p21.2) and consists of 17 exons. BAP1 encodes a nuclear ubiquitin carboxy-terminal hydrolase (UCH), one of several classes of deubiquitinating enzymes. Human BAP1 protein (80.4 kDa) is 729 amino acids long and has three domains: A, a ubiquitin carboxyl-terminal hydrolase (UCH) N-terminal catalytic domain, which removes ubiquitin from ubiquitylated substrates; B, a unique linker region, which includes a host cell factor C1 binding domain at residues 356–385; C, a C-terminal domain, including residues 598–729, which include a UCH37-like domain (ULD) at residues 675–693 and two nuclear localization sequences at residues 656–661 and 717–722 [17].

BAP1 is a nuclear-localized deubiquitinating enzyme with ubiquitin carboxyl hydrolase activity and two nuclear localization signal motifs [11,15,16,17]. It has also been speculated that the BAP1 gene is located on chromosome 3p21.1, a genomic region frequently mutated in a variety of human malignancies [18,19] (Figure 2). 

Somatic alterations affecting BAP1 were observed in familial mesotheliomas, indicating biallelic inactivation [16,19]. Germline mutations in BAP1 were discovered in two families with a high incidence of mesothelioma and an elevated risk of malignancies, such as cutaneous melanoma, renal cell carcinoma, cholangiocarcinoma, and basal cell carcinoma [16,18,19]. Mutations in BAP1 occur in 5–15% of sporadic ccRCC tumors, and germline BAP1 mutations occur in some cases of ccRCC [16,17,18,19]. 

**Figure 3:**
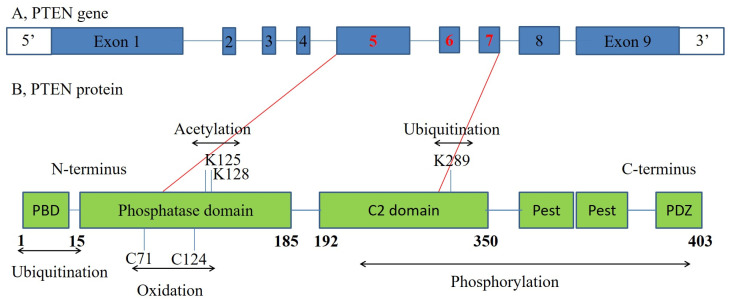
PTEN gene structure (**A**) and PTEN protein structure (**B**). The tumor suppressor gene PTEN, which maps to 10q23.31, encodes for a protein with 403 amino acids. The protein has 2 major functional domains, the N-terminal (encoded by exon 1–5) and the C-terminal domains (encoded by exon 6–9). The protein is phosphatidylinositol-3,4,5-trisphosphate 3-phosphatase. The phosphatase domain (encoded by exon 5) contains the active site, which carries out the enzymatic function of the protein, while the C2 domain binds the phospholipid membrane.

PTEN is a tumor suppressor gene on chromosome 10q23.3 that encodes a 403 amino acid dual-specificity lipid and protein phosphatase (Figure 3) [20,21,22,23]. 

The PTEN dephosphorylates PI-(3,4,5)-triphosphate, regulating the PI(3)K-AKT-mTOR pathway, and leading to G1 cell cycle arrest and apoptosis, which inhibits cell migration [24,25,26,27,28,29,30,31,32,33]. Germline PTEN mutations have been detected in patients with autosomal dominant cancer predisposition syndromes, such as Cowden disease, Bannayan–Zonana syndrome, and Lhermitte–Duclos disease [25,26]. The frequent somatic mutations caused by this tumor suppressor gene have even been reported in a variety of sporadic tumors, including endometrial cancer, breast cancer, prostate cancer, malignant melanoma, and thyroid tumors [24,27,28,29,30,31]. In contrast to these tumors, PTEN mutations have rarely been reported in non-small cell lung cancer and gastric cancer [28,33]. PTEN loss may predispose an individual to colorectal neoplasia and polyposis [26]. Moreover, PTEN may play a prognostic role in the development and malignant transformation of soft tissue sarcoma [33]. Some PTEN deletions and mutations were observed in sporadic kidney cancers. An aggressive phenotype in some types of tumors is reported to be associated with the alteration in this gene [27,28,29,31]. 

Unfortunately, there is still no clear evidence regarding the genetic background of the development of renal tumors, and no correlation has been found so far between the genetic alterations mentioned above and the clinicopathological status, prognosis, or outcome of the patients. 

In this study, using a sequencing approach, we analyzed the mutations of VHL, PTEN, and BAP1 in 24 renal tumor specimens. The VHL gene was sequenced, and we aimed to report the somatic mutations we found. Although the prognostic impact of the PTEN and BAP1 mutations in renal carcinoma is under investigation, the prognostic significance of mutated PTEN and BAP1 genes has not ever been fully described in RCC. In the literature, we found hot spot exons of PTEN and BAP1 genes that were detected in other carcinomas such as endometrial carcinoma [17,27]. Therefore, the present study aimed to detect mutations only in the hot spots. Our purpose was also to establish the role of BAP1 as a tumor suppressor protein in the tumorigenesis of renal cancer in the examined group of patients. We also associated the presence of every identified genetic alteration with the clinicopathology of the examined patients.

## 2. Materials and Methods

### 2.1. Study Population and Preparation of Renal Tumor Tissue Samples from Patients

The study included a cohort of patients (24 patients in total) with renal tumors who underwent surgical resection at the Department of Urology, University of Debrecen. All patients gave informed consent before participating in the study. The study was approved by the Ethics Committee of the University of Debrecen (UD REC/IEC 4831-2017). Tumors were staged using the TNM staging system of the Union for International Cancer Control. Histological grade was determined according to World Health Organization criteria. The local invasion was assessed using T staging, and lymphatic status was recorded as positive or negative. After confirming the presence of the renal neoplasm on cryosections in each case, the tumor tissues were immediately frozen in liquid nitrogen and stored at −80 °C until further molecular processing. The rest of the surgical specimens were processed routinely for morphological analytical histopathology to establish the final tumor diagnosis in each case of the formalin-fixed paraffin sections, including the histotype, the grade, the pathologic staging (pT), and lymph node involvement, using the internationally approved standard protocols for RCC (see above). All human tissue samples originated from primary tumors without metastases. 

### 2.2. RNA Isolation and Reverse Transcription PCR

Homogenization of the kidney tissue samples was carried out with Tissue Ruptor (IKA^®^-WERKE GmbH, Staufen im Breisgau, Germany). Total RNA from tumor tissues was isolated using a NucleoSpin DNA/RNA/Protein Kit (Macherey-Nagel, Düren, Germany) according to the manufacturer’s instructions. Quantitative and qualitative assays for RNA were performed using a Nanodrop ND-1000 UV Spectrophotometer (Nanodrop Technologies, Wilmington, DE, USA). 

Reverse transcription of RNAs from each sample into cDNA was carried out by using a Tetro cDNA Synthesis Kit (Bioline Reagents, London, UK) according to the manufacturer’s instructions. RT-PCR reaction for the detection of the PTEN and BAP1 genes in kidney tissue samples was performed using 25 μL of the final reaction volume with gene-specific primers (The primer sequences for BAP1 forward: CCCGCGGGAAGATGAATAA, reverse: ACCCCCTTGACACCGAAATC; for PTEN forward: TGGATTCGACTTAGACTTGACCT-3′, reverse: 5′-GGTGGGTTATGGTCTTCAAAAGG-3). 

The MCF-7 human breast cancer cell line was used as a positive control for the detection of PTEN and BAP1 genes. The RT-PCR reaction consisted of 35 cycles (95 °C for 15 s, 60 °C for 30 s, 72 °C for 10 s) and lasted for a 2 min extension at 72 °C. β-actin was used as a positive internal control. PCR products were separated in a 1.5% agarose gel containing GelRed and detected under UV light, digitalized with AlphaDigiDoc™ RT (Alpha Innotech, Santa Clara, CA, USA). To determine the size of DNA, a 50 bp DNA marker (Bioline Reagents) was applied.

### 2.3. Tissue Sample Preparation and DNA Isolation

Human tissue samples obtained from 24 patients with renal tumors at the time of surgery were also used for DNA isolation. Sample genomic DNA was extracted from whole-tumor tissues using a Nucleospin DNA isolation kit (Macherey-Nagel, Düren, Germany). DNA was qualified using a Nanodrop ND-1000 UV Spectrophotometer (Nanodrop Technologies, Wilmington, DE). DNA samples with an optical density value higher than 1.9 at 260/280 nm were used for further sequencing analyses.

### 2.4. PCR Amplification Prior to Sequencing

*A.*  *for VHL*

The three exons of the VHL gene were amplified using 4 intron-based primer pairs. Exon 1 was amplified with two overlapping primer pairs. Primers for the exons were as follows: Exon 1A: forward: 5′-TAT AGT GGA AAT ACA GTA ACG AG-3′ and reverse: 5′-GAA GTT GAG CCA TAC GG-3′; Exon 1B: forward: 5′-AGA GTA CGG CCC TGA AGA A-3′ and reverse: 5′-GCT TAC GAG CAG CGT ACA-3′; Exon 2: forward: 5′-ATC TCC TGA CCT CAT GAT CC-3′ and reverse: 5′-GGG CTT AAT TTT TCA AGT GG-3′; Exon 3: forward: 5′-TGA GAT CCA TCA GTA GTA CAG G-3′ and reverse: 5′-CTA AGG AAG GAA CCA GTC C-3′. Each 50-µL reaction mixture for VHL amplification contained 100 ng of genomic DNA, 0.2 mM of each deoxynucleotide triphosphate, 1 × Green GoTaq^®^ Reaction Buffer, 2.5 mM MgCl_2_, 1.25 unit of GoTaq^®^ DNA Polymerase (Promega), and 10 pmol/µL of each primer. PCR reactions were performed with the following programs: denaturation at 95 °C for 10 min, followed by 40 cycles at 95 °C for 1 min, 56 °C (exon 3) and 59 °C (exon 1A, 1B, 2) for 1 min, 72 °C for 1 min, and final extension at 72 °C for 7 min.

*B.*  *for PTEN*

The three exons of the PTEN gene were amplified using 4 intron-based primer pairs. Exon 5 was amplified with two overlapping primer pairs. Primers for the exons were as follows: Exon 5: forward: 5′-AGT TTG TAT GCA ACA TTT CTA A-3′ and reverse: 5′-TTC CAG CTT TAC AGT GAA TTG-3′ (first pair) and forward: 5′-GAC CAA TGG CTA AGT GAA GAT-3′ and reverse: 5′-AGC AAC TAT CTT TAA AAC CTG T-3′ (second pair); Exon 6: forward: 5′-TTG GCT TCT CTT TTT TTT CTG-3′ and reverse: 5′-ACA TGG AAG GAT GAG AAT TTC-3′; Exon 7: forward: 5′-ACA GAA TCC ATA TTT CGT GTA-3′ and reverse: 5′-TAA TGT CTC ACC AAT GCC A-3′ [25]. Each 50-µL reaction mixture for PTEN amplification contained 100 ng of genomic DNA, 0.2 mM of each deoxynucleotide triphosphate, 1 × Green GoTaq^®^ Reaction Buffer, 2.5 mM MgCl_2_, 1.25 units of GoTaq^®^ DNA Polymerase (Promega), and 10 pmol/µL of each primer. PCR reactions were performed with the following programs: denaturation at 94 °C for 10 min, followed by 35 cycles of 94 °C for 30 s, 55 °C for 30 s, 72 °C for 30 s, and final extension at 72 °C for 10 min.

*C.*  *for BAP1*

The four exons of the BAP1 gene (exons 14–17) were amplified using 6 intron-based primer pairs. Exons 15–16 were amplified with one primer pair. Primers for the exons were as follows: Exon 14: forward: 5′-CCTTGGACTGGCTCACTGG-3′ and reverse: 5′-CAGCCACCAATCTTCACACC-3′; Exons 15–16: forward: 5′-CTCGTGGGGCTTTGTTGC-3′ and reverse: 5′-AGGGGAGGGGAGCTGAAG-3′; Exon 17: forward: 5′-ATGCGCTGCTGTCTTAACTG-3′ and reverse: 5′-ACTGGGAAAAGGGGAAGTGG-3′. Each 50-µL reaction mixture for BAP1 amplification contained 100 ng of genomic DNA, 0.2 mM of each deoxynucleotide triphosphate, 1 × Green GoTaq^®^ Reaction Buffer, 2.5 mM MgCl_2_, 1.25 unit of GoTaq^®^ DNA Polymerase (Promega), 10 pmol/µL of each primer. PCR reactions were performed with the following programs: denaturation at 95 °C for 10 min, followed by 40 cycles of 95 °C for 1 min, 60 °C for 1 min, 72 °C for 1 min, and final extension at 72 °C for 7 min.

### 2.5. DNA Sequencing

The size of the PCR products was checked using a 1.5% agarose gel. The products were purified (DyeEx Spin Kit, Qiagen) and concentrated (Vacuum Concentrator 5301, Eppendorf, Hamburg, Germany). 

Forward and reverse direct fluorescent sequencing (conventional Sanger sequencing) of PCR products were performed using an ABI PRISM 3130 DNA sequencer (Perkin-Elmer, Foster City, CA, USA) and the BigDye Terminator v.1.1 Cycle Sequencing Kit (Applied Biosystems). The sequences were analyzed using Finch TV software version 1.4.0 (Geospiza Inc., Seattle, WA, USA). The obtained sequences were compared with the NCBI Reference Sequence: NM_000551.3 in the case of the VHL gene, NCBI Reference Sequence: NM_004656.2 in the case of the BAP1 gene, and with the NCBI Reference Sequence: NM_000314.4 in the case of the PTEN gene. Mutations were identified based on information from the Human Gene Mutation Database (HGMD).

## 3. Results

### 3.1. Clinicopathological Characteristics of the Patients 

In our study, 24 human kidney tumor samples were investigated. The clinicopathological data of the patients are shown in Table 1.

Twelve patients were males (50%) and twelve patients were females (50%) with a median age of 54 years (range 36–72). Patients with a previous or secondary malignancy, uncontrolled or serious infection, and those who had undergone radiation therapy, chemotherapy, or immunotherapy were not recorded at the time of the surgery. Patients included in the study did not receive any neoadjuvant therapy before surgery. The surgery procedures performed as a curative treatment for the patients were right or left kidney resection and laparoscopic right or left nephrectomy. According to histological examination among the examined 24 cases, 20 presented a clear cell histotype of RCC (ccRCC), 2 cases presented angiomyolipoma, 1 case was identified as renal oncocytoma, and 1 case related to papillary renal carcinoma (pRCC) type. According to the TNM staging system and the grade classification, 8 cases (38.095%) were classified with G2, and 13 cases (61.53%) belonged to the G3 group of the RCC type (Table 2). Information about patients’ smoking habits was not available. 

### 3.2. Expression of mRNA for BAP1 and PTEN in Human Kidney Tumor Tissue Samples

In order to examine the expression pattern of mRNA expression in BAP1 and PTEN genes in human kidney tumor tissues, RT-PCR was performed on all of the samples collected for the study. Template-free and reverse transcriptase-free controls eliminated non-specific amplification and DNA contamination, respectively. PCR amplification with specific primers for β-actin produced a single product in every sample, confirming the absence of RNA degradation in the samples. As a positive control, a human breast cancer cell line (MCF-7) was used. 

**Figure 4:**
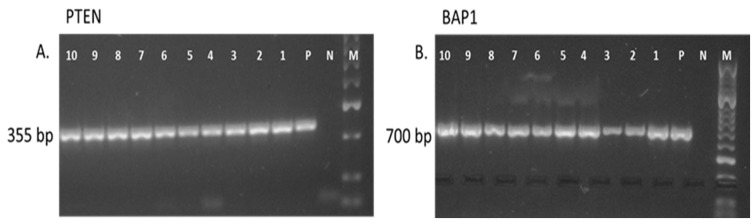
Representative RT-PCR analysis of mRNA for PTEN and BAP1 in 10 human kidney tumor specimens and the MCF-7 breast cancer cell line (**A**,**B**). The size of the PCR products was 350 bp, as expected for PTEN (**A**), and 700 bp, as expected for BAP1 (**B**). Lane M, molecular marker (50-bp DNA ladder); Lane P, positive control (MCF-7 cell line); Lane N, no template control; Lanes 1–10 (**A**,**B**), representative human kidney cancer specimens.

PCR amplification derived from the kidney tumoral tissues using the oligonucleotide primers for human BAP1 and PTEN genes yielded the size of 350 bp for PTEN and 700 bp for the BAP1 genes (Figure 4A,B). The expression of the BAP1 and PTEN genes was detected in all of the 24 primary kidney tumor tissue samples examined. A representative picture of the expression of the PTEN and BAP1 genes is shown in Figure 4A,B. 

By using a cohort of fresh frozen renal tissue samples obtained from 24 patients undergoing surgical resection, we analyzed the mutations of the VHL gene, the BAP1 gene in exons 14–17, and the PTEN gene in exons 5–7. These results are shown point by point in the following sections.

### 3.3. VHL Mutations and IVS1-195 Nt G/A Polymorphism in Human Renal Tumor Specimens Examined

**Figure 5:**
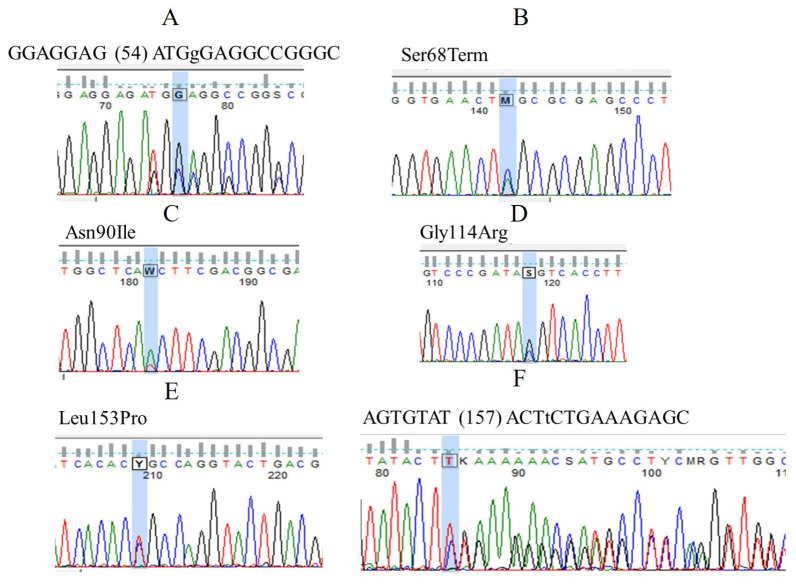
Electropherograms representing VHL mutations: (**A**) GGAGGAG (54) ATGgGAGGCCGGGC heterozygote; (**B**) Ser68Term heterozygote (TCG-TAG); (**C**) Asn90Ile heterozygote (AAC-ATC); (**D**) Gly114Arg heterozygote (GGT-CGT); (**E**) Leu153Pro heterozygote (CTG-CCG); (**F**) AGTGTAT (157) ACTtCTGAAAGAGC heterozygote. Based on The Human Gene Mutation Database (HGMD), all 6 mutations are pathogenic and cause Von Hippel–Lindau syndrome.

All the samples involved in the study were screened for mutations in the VHL gene using direct sequencing. In our study, six different VHL mutations (three missense mutations, one nonsense mutation, and two small insertions) were confirmed in the 24 patients studied. One of the samples had the IVS1-195 nt G/A polymorphism in homozygous form with the GGAGGAG (54) ATGgGAGGCCGGGC mutation (Figure 5). 

Three of the six identified mutations were located in exon 1, two in exon 2, and one in exon 3. The identified mutations were the following: In exon 1: 1, GGAGGAG (54) ATGgGAGGCCGGGC in heterozygous form: this is an insertion of a guanine at position 54; 2, Ser68Term in heterozygous form: this is a nonsense point mutation, where the serine-68 residue changed to stop codon; 3, Asn90Ile heterozygous form: this is a missense mutation with amino acid change (asparagine to isoleucine) at position 90 [32,33]. In exon 2: 4, Gly114Arg in heterozygous form: this is a missense mutation with an amino acid change (glycine to arginine) at position 114 [34]; 5, Leu153Pro in heterozygous form: this is a missense mutation with an amino acid change (leucine to proline) at position 153 [35]. In exon 3: 6, AGTGTAT (157) ACTtCTGAAAGAGC in heterozygous form: this is an insertion of a thymine at position 157 [36]. 

**Figure 6:**
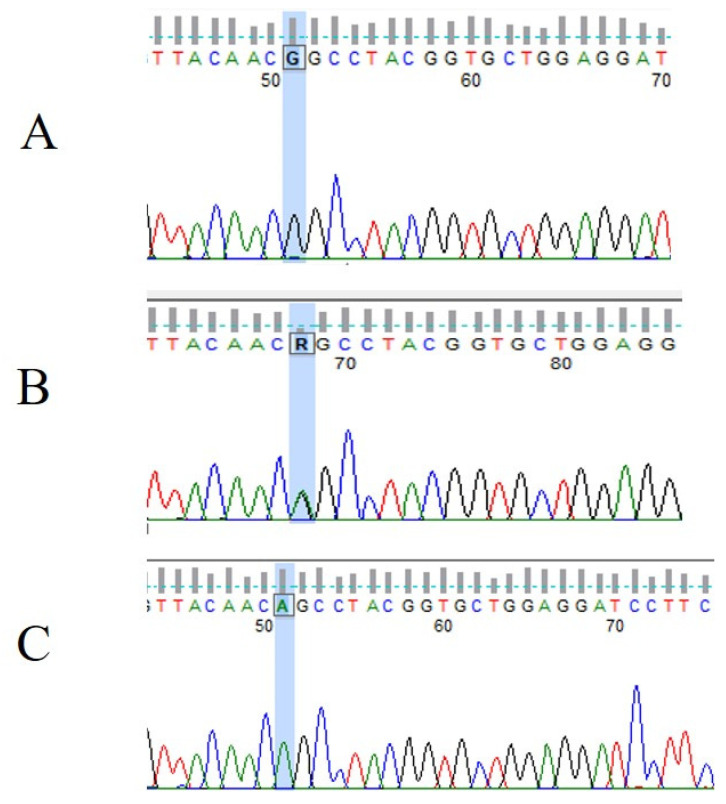
Electropherograms representing (**A**) wild type (n = 2), (**B**) heterozygote type (n = 9), and (**C**) homozygote (n = 13) type of VHL IVS1-195 nt G/A (rs779805) polymorphism.

IVS1-195 nt G/A polymorphism was confirmed in heterozygote form in 9 cases and homozygote form in 13 cases. This VHL polymorphism (rs779805) is a G/A variation at the 195 nucleotide position before exon 1 in the intron region of the VHL gene [37]. Electropherograms representing the wild type and heterozygote and homozygote types of the VHL IVS1-195 nt G/A polymorphism are shown in Figure 6.

### 3.4. PTEN Mutations and Polymorphism in Human Renal Tumor Specimens Examined

**Figure 7:**
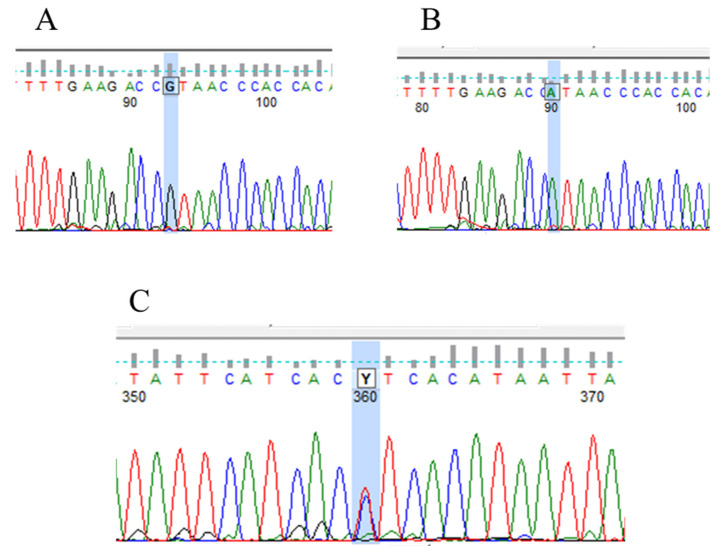
Electropherograms representing PTEN mutation and polymorphism (**A**) His93Arg homozygote mutation (CAT-CGT); (**B**) wild type; (**C**) IVS5 + 217 nt C/T polymorphism.

In our study, one PTEN mutation and one polymorphism were confirmed in 24 unrelated patients. The PTEN mutation (p.His93Arg) was detected in exon 5 in homozygous form. It was a point mutation with an amino acid change (histidine to arginine) at position 93. According to the HGMD database, the His93Arg mutation is pathogenic and can cause autism spectrum disorder and macrocephaly [38]. The IVS5 + 217 nt C/T polymorphism was detected after exon 5 at position + 217. It is a C/T variation in heterozygous form (Figure 7). 

### 3.5. Wild Types of the BAP1 Gene in Human Renal Tumor Specimens Examined

Our samples were sequenced only for the hot spot exons of the BAP1 gene (exon 14–17). The mutation was not observed inside of the hot spot exons (14–17) of the BAP1 gene in any of the samples analyzed. 

### 3.6. Relationship between VHL, PTEN, and BAP1 Mutations and Clinicopathological Features of the Human Specimens Examined

Basic clinical data of the patients concerned either with the VHL or PTEN mutations are summarized in Table 3. 

Within the group of these patients, there were six females and two males. Six of the patients were relatively young, and the age of these patients ranged from 36 to 57 years, with two patients being 72 years old. At the time of the first observation, the tumors had already developed into a large size. The size of the tumors ranged from 3 cm to 10 cm in the largest diameter. In patients with the VHL mutation, other types of tumors (cervical carcinoma and bladder cancer) were observed in two cases. Regarding the patients with VHL mutations, in three cases, the histological type displayed a type of clear cell renal cell carcinoma that was Grade 2 and Grade 3. One patient with VHL mutation represented angiomyolipoma and oncocytoma (oxyphilic adenoma). One of the patients with the VHL mutation developed metastasis of the pancreas (7 years after the primary kidney tumor) and was treated with Sunitinib (Sutent). In one patient characterized with PTEN mutation, small foci of papillary architecture were present (pRCC). No mutations in the BAP1 gene (exon 14–17) were observed in any of these patients. The BAP1 gene (exon 14–17) showed wild type in any of the cases.

## 4. Discussion

Since human renal cell cancer is a heterogeneous disease, a large morphological and molecular inter-tumor heterogeneity may occur within the same histotype. In addition, intra-tumor heterogeneity can also be detected in the primary and metastatic lesions of the same patient. Mutations in some genes such as VHL1, PTEN, JAK3, and TP53 have already been recognized; however, specific cases can also be found with these types of genetic alterations. These mutations contribute highly to the development of the disease and the clinical outcome of the patient [2,3,4,39,40]. According to the Cancer Genome Atlas database, the most frequent somatic mutations in RCC mainly include changes in the VHL gene, followed by alterations of the PI(3)K/AKT/mTOR pathway, thus affecting the response of the patient to the therapy [7,8,10]. In familial RCC, as well as in sporadic ccRCC, it is common that one VHL allele is deleted, whereas the remaining allele acquires an inactivating aberration, rendering a complete loss of functional protein [7,8,10]. Mutational inactivation of the VHL tumor suppressor gene causes an inappropriate accumulation of the hypoxia-inducible transcription factor (HIF), which promotes tumorigenesis in the context of kidney cancer [9]. Renal angiomyolipoma is the most common form of renal disease in patients affected by mutations in the tuberous sclerosis complex (TSC) [41]. Previous studies have found that the missense mutation of the tuberous sclerosis complex 1 (TSC1) and the mutation of tuberous sclerosis complex 2 (TSC2) encoding tRNA-specific adenosine deaminase 1 (TAD1) showed a correlation with a high risk of renal AML [41]. Though VHL mutation is not common in AML, a study of the Kashmirian population surprisingly described VHL mutations in one of the non-clear-cell RCC samples that were identified as AML [42]. Genetic testing highlighted partial VHL gene deletion in a case study of a 63-year-old man with bilateral and multifocal renal oncocytoma. Partial VHL gene deletion suggests a higher risk of renal cell carcinoma than that seen in patients with complete deletions of the VHL gene [43]. 

The clinicopathological impacts of somatic VHL mutations or promoter methylation have been studied in a variety of cases involving RCC [10]. Some investigators have published results that suggest a significant association of VHL-altering events with pathological or survival outcomes; moreover, the presence of VHL alteration significantly correlated with a standard prognostic factor and with the stage of the disease in patients with RCC [10]. 

It has also been speculated that additional tumor suppressors are present on chromosome 3p, as the loss of this arm is a frequent event in ccRCC and other tumors [17]. BAP functions as a de-ubiquitinating enzyme that regulates multiple cellular pathways related to tumorigenesis in renal cell carcinomas [15]. Mutations in BAP1 (3p21.31) have been shown to be associated with poor cancer-specific survival in renal carcinoma [44,45]. Recently, BAP1 gene mutations have been reported in 14% of ccRCCs [12,15,19]. 

PTEN is one of the most frequent genes mutated in cancers. It was identified on chromosome 10q23.3, which is homozygously deleted in many human malignancies. For instance, PTEN is mutated/deleted in 27–44% of glioblastomas, 43–50% of prostate carcinomas, and 34–50% of endometrial carcinomas [16]. Using single-strand conformation, polymorphism analysis, and DNA sequencing, PTEN mutation revealed factors of mutation outside of the exons 5–7 of PTEN in endometrial carcinoma. Grades 1–2 were significant and independent prognostic indicators for favorable survival. Mutations in exons 5–7 were associated with a poor prognosis. No significant correlation was observed between PTEN mutations outside exons 5–7 and clinicopathological features. These findings suggest that only mutations outside of exons 5–7 of PTEN might represent a molecular predictor of favorable survival, independent of clinical and pathological characteristics of tumors [17].

In the kidneys, the tumor suppressor gene PTEN is a ubiquitously expressed epithelial cell-enriched phosphatase that is located in the cytoplasm of epithelial cells. The loss of the tumor suppressor protein PTEN during renal carcinogenesis has also been analyzed. PTEN expression is nearly lost during the carcinogenesis of ccRCCs, suggesting an important role in tumorigenesis. An earlier study showed a loss of membrane localization during the carcinogenesis of ccRCC [46]. The disturbance of all membrane-associated activities of PTEN can be expected, leading to a disturbance in the PIP3/Akt signaling pathway. An aggressive phenotype in renal cancer has been reported to be associated with alterations in the PTEN gene [46]. 

The prognostic significance of PTEN mutations is also under investigation regarding RCC. However, a limited number of recent studies have shown that PTEN mutation is associated with a favorable prognosis in patients with this disease; moreover, no studies have ever described the prognostic significance of the mutated exons of PTEN in renal cancer. 

In the present study, we investigated the expression of mRNA for BAP1 and PTEN genes in 24 renal tumor specimens. Fifty percent of the tissue specimens originated from men and the other fifty percent from women. The expression of mRNA for BAP1 and PTEN was found in all the samples investigated. These results might suggest that both of these genes may have a tumor suppressor function in the development of renal carcinoma [46,47]. 

In this study, we have also provided one of the few available reports on the mutation analysis of the VHL (exons 1–3), BAP1 (exons 14–17), and PTEN (exons 5–7) genes in RCC using a Sanger sequencing platform. The current study investigated mutations of these genes and the prognostic impact of mutated hot spot exons. The relationship between mutations and clinicopathological characteristics was also examined in the present study.

VHL mutations occurred in 25% of the examined specimens. We found mutations in exons 1–3 of the VHL gene. Of the six VHL mutations, three were missense mutations, one was a nonsense mutation, and two were small insertions. Based on data from the HGMD, all six mutations cause Von Hippel–Lindau syndrome. IVS1-195 nt G/A polymorphism was confirmed in heterozygote form in 9 cases and in homozygote form in 13 cases. Three of the patients identified with VHL mutations belonged to the ccRCC histotype, two were classified as angiomyolipomas, and one was described as oxyphilic adenoma. These results are in contrast with the earlier opinion that VHL mutations are exclusively restricted to ccRCC and similar to the results described in previous studies [43]. Teh et al. reported a case of a patient with bilateral multiple oncocytomas and cysts associated with a constitutional translocation and a rare constitutional VHL missense substitution [48].

Regarding the mechanism of the mutations, in our cases, we determined that, in the mutation of the VHL Ser68Term, a premature stop codon can yield a truncated abbreviated protein product, which is predicted to lead to a loss of VHL protein function [34]. The mutation type of the VHL Asn90Ile confers a loss of function to the VHL protein, as demonstrated by the failure to regulate HIF-1α ubiquitination and degradation [35]. Alterations of VHL such as Gly114Arg and Leu153Pro lie within the CCT complex-binding region of the VHL protein. Gly114Arg is predicted to confer a loss of function to the VHL protein, as indicated by the failure to bind to the CCT complex [36]. Leu153Pro has not yet been biochemically characterized in the HGMD database; thus, its effect on VHL protein function is unknown [37]. In our study, we also identified two insertion VHL mutants at positions 54 and 157, with the addition of a guanine and a thymine, respectively. This may result in a frame shift that changes the reading of subsequent codons and alters the entire amino acid sequence that follows the mutation [34,35,36].

Literature-based functional analyses suggest that all six VHL mutations in the clinical cases we examined most probably lead to pVHL loss. Mutational inactivation of the VHL tumor suppressor gene causes an inappropriate accumulation of HIF, which promotes tumorigenesis in the context of kidney cancer [8]. Presumably, the process of angiogenesis in primary renal tumors occurs only in the early stage. However, from the tumor size of the patients, we might suggest that the detected type of VHL mutations can positively influence the growth of the tumor. The largest case of oncocytoma (10 cm), which developed in a 72-year-old female patient, definitely deserves attention.

One patient’s tumor metastasized, and two of the patients died 5–6 years after surgery. However, we did not find any significant association between histotype, clinicopathological background, and the VHL mutation variants observed in our study.

Based on the literature, the point mutation of the VHL gene occurs in about 60% of cases; moreover, large deletion occurs in about 40% of patients with RCC [10,47,49,50]. The mutation of the VHL gene was found to be a frequent event in clear cell, granular, and sarcomatoid renal carcinomas, but not in papillary renal carcinomas. Cai Lv et al. analyzed rs779805 VHL polymorphism, and their results indicated that the G allele could slightly increase the risk of RCC [39].

Other studies showed that VHL gene mutations result in the inactivation of this gene, which has been observed in major conventional RCC [47]. Comparing our results with earlier observations, we may conclude that missense mutations are probably the most common type among all of the mutations, which may result in the inactivation of the VHL gene and a loss of protein function [10]. Nevertheless, several studies have reported that loss-of-function mutations (LOF), rather than other types of VHL alteration, showed a meaningful correlation with survival [10]. However, other studies did not observe a significant correlation between LOF mutations and survival. Interestingly, LOF mutations acted as good predictive markers for VEGF-targeted therapy in patients with RCC. Patients with LOF mutations obtained a significantly higher response rate than those with VHL wild-type tumors [10]. Tumor or cyst development in VHL disease is linked to somatic inactivation or the loss of the remaining wild-type VHL allele [51].

In the case of the BAP1 gene, all of the samples were wild type in our study. The mutations of four exons of the BAP1 gene (exons 14–17) were examined in all of the kidney cancer samples studied. BAP1 mutation was not observed in any of the renal tumor samples studied. According to the literature [51], Sanger sequencing is very reliable and reproducible in detecting BAP1 point mutations and small deletions. However, this technique would likely not be able to detect large deletions. This could be one of the reasons why we did not find any mutations within the examined hot spot exons 14–17 in our renal cancer tissue specimens. For accurate detection of the deletions of large exons, several Multiplex Ligation-dependent Probe Amplification (MLPA) probes have to be used [15,17].

Minardi published the first report in which BAP1 was studied in pT1 ccRCC tumors [15]. None of the samples from patients with pT1 ccRCC showed a total loss of nuclear BAP1 staining [15]. A significant negative correlation was shown between nuclear BAP1 expression and tumor size and between nuclear BAP1 expression and grade. Nuclear BAP1 staining was not correlated with disease-specific 5-year survival. Immunohistochemistry is the most reliable method to detect BAP1 nuclear loss in ccRCC [15,16,17].

In our investigation, PTEN mutation was observed only in one of the samples, and another sample was characterized using genetic polymorphism. According to the HGMD database, the mutation observed in our study (His93Arg) causes autism spectrum disorder and macrocephaly [44]. This sequence change replaces histidine with arginine at position 93 of the PTEN protein. The histidine residue is highly conserved, and there is a small difference between histidine and arginine. This variant has been reported in an individual with PTEN hamartoma tumor syndrome and individuals affected with macrocephaly and autism [46]. Patients with PTEN polymorphism were identified with ccRCC in the G3 pathological stage, and the patient with PTEN mutations was classified as papillary RCC with G2.

The PTEN p.His93Arg mutation (in exon 5) was found in a 46-year-old male patient with papillary RCC with pathological Grade 2 and at a T1, N0, M0 clinical stage. The tumor was already well developed (5 cm). This mutation was detected within the phosphatase domain of the gene, near the active site, which carries out the enzymatic function of the protein. Probably, the consequence of this mutation of the PTEN gene resulted in a decrease in the phosphatase activity of PTEN. The dephosphorylation and inactivation of the focal adhesion kinase (FAK) involves the protein phosphatase activity of PTEN. This mechanism connects the interaction between the extracellular matrix and the cytoskeleton. PTEN is a negative regulator of the integrin-mediated invasion [40,46]. We also assume that this type of PTEN mutation leads to the inactivation of the PI3K/AKT pathway and may promote tumorigenesis [32].

The IVS5 + 217 nt C/T polymorphism of PTEN was detected after exon 5 at position + 217. According to the HGMD database, it is a benign C/T variation (rs35560700) in heterozygous form; thus, we assume that the development and prognosis of the disease in the affected patient are not exclusively associated with these types of genetic alterations.

However, there was no significant association between PTEN expression, histotype, and histological grading observed in the present study. Nevertheless, our data might suggest that the loss of the tumor suppressor PTEN is probably an early event in the carcinogenesis of RCC [46].

The relatively limited number of cases and the even smaller number of patients with available primary tumors and metastases do not allow us to draw definitive conclusions on the role of the VHL, PTEN, and BAP1 mutations in renal carcinogenesis. However, based on our results, we can conclude that these mutations are non-mutually exclusive and hit a variable number of loci. In addition, in our mutational analysis, we may have missed mutations in the genes outside of the investigated exons of the genes mostly affected by hot spot mutations. We may also conclude that the outcome of patients with RCC-identified genetic aberrations is probably associated with losses at chromosomes 3 and 10; however, the overall mutation rate was low in all of the samples involved in the analyses, and the development and prognosis of the disease are not associated with these types of genetic aberrations. To clarify the intra-tumor heterogeneity of genetic alterations in RCC, larger studies covering all the RCC histotypes with potential paired samples of primary tumors and metastases are required.

In conclusion, this study indicates that VHL, PTEN, and BAP1 gene alterations are not significantly associated with the pathological features or survival of patients with RCC. Large-scale studies are needed to reveal the predictive or prognostic roles of the mutational subtypes of these genes in patients with RCC.

## 5. Conclusions

Based on our studies, we assume that changes in VHL and PTEN genes are not significantly related to the pathological characteristics or survival of patients with renal tumors. All the detected VHL mutations examined lead to the loss of pVHL, thus affecting further processes in tumorigenesis. The described genetic polymorphism (rs779805) of VHL is also considered to be pathogenic and may contribute to the development of renal tumors. VHL mutation is rare in AML; however, in our study, two young female patients with AML harbored alterations of the VHL gene. VHL mutation was also observed in a 72-year-old woman diagnosed with oncocytoma. In our study, we report, for the first time, a PTEN His93Arg mutation in a patient with papillary RCC. Overall, we assume that the loss of VHL and PTEN may cooperatively contribute to the accelerated progress of renal cancer. However, BAP1 seems to be uninvolved in the reported tumorigenesis. In conclusion, we assume that the outcome of the patients with renal tumor-identified genetic aberrations can be associated with losses at chromosomes 3 and 10. As the overall mutation rate of the VHL, PTEN, and BAP1 genes was low in all samples investigated, the development and prognosis of the disease in the examined patients were not exclusively associated with these types of genetic alterations.

## Figures and Tables

**Table 1:** Clinicopathological data of renal tumor specimens.

Sample Number	Age/Gender	Histological Type	Histological Grade	Pathological Stage	TNM Classification
1.	40/female	ccRCC	2	pT1a	T1, N0, M0
2.	68/female	ccRCC	2	pT4	T4, N0, M0
3.	62/male	ccRCC	3	pT4	T4, N0, M0
4.	64/male	ccRCC	3	pT4	T4, N0, M0
5.	72/female	ccRCC	3	pT3a	T3, N0, M0
6.	55/male	ccRCC	2	pT1a	T1, N0, M0
7.	57/female	ccRCC	2	pT1a	T1, N0, M0
8.	36/male	ccRCC	3	pT4	T4, N0, M0
9.	39/female	ccRCC	3	pT1a	T1, N0, M0
10.	72/female	AML	―	―	―
11.	72/female	oncocytoma	―	―	―
12.	47/female	ccRCC	3	pT1a	T1, N0, M0
13.	69/male	ccRCC	3	pT4	T4, N0, M0
14.	53/female	ccRCC	3	pT4	T4, N0, M0
15.	49/male	ccRCC	3	pT1a	T1, N0, M0
16.	52/male	ccRCC	3	pT1b	T1, N0, M0
17.	56/male	ccRCC	3	pT1b	T1,Nx, Mx
18.	69/male	ccRCC	3	pT1a	T1, N0, M0
19.	37/female	ccRCC	3	pT1b	T1, N0, M0
20.	49/male	ccRCC	2	pT1a	T1, N0, M0
21.	72/female	ccRCC	2	pT1a	T1, N0, M0
22.	36/female	AML	―	―	―
23.	41/male	ccRCC	2	pT1b	T1, N0, M0
24.	46/male	pRCC	2	pT1b	T1, N0, M0

T1a: tumor size is less than 4 cm and organ localized, pT1b: tumor size is more than 4 cm but less than 7 cm, and the tumor is organ localized; pT3a: tumor extends into the renal vein or its segmental branches, invades the pelvicalyceal system, or invades perirenal and/or renal sinus fat, albeit not beyond Gerota’s fascia, pT4: tumor is extended to adrenal gland, ccRCC: clear cell renal cell carcinoma, pRCC: papillary type of renal carcinoma, AML: angiomyolipoma. N0/M0: lymph node status of the patient is negative and there are no metastases; Nx/Mx: lymph node status of the patient is unknown.

**Table 2:** Histological grades and genders of the 21 patients analyzed.

Histological Grade	Grade %	Men Number %	Women Number %
Grade 2	8 cases (38.095%)	4 50.0	4 50.0
Grade 3	13 cases (61.90%)	8 61.53	5 48.46

**Table 3:** Clinicopathological status of patients with VHL and PTEN mutations.

Sample Number	Age/Gender	Histological Type	Histological Grade	Pathological Stage	TNM Clinical Stage	Tumor Size	Surgery Type	Mutation	Other Tumors
1.	72/female	Oncocytoma	―	―	―	10 cm	right site radical nephrectomy	VHL mutation	was not observed
2.	57/female	ccRCC	2	pT1a	T1, N0, M0	3.3–4 cm	right site kidney nephrectomy	VHL mutation and VHL polymorphism	was not observed
3.	36/male	ccRCC	3	pT4	T4, N0, M0	4 cm	right site kidney resection	PTEN polymorphism	was not observed
4.	39/female	AML	―	―	―	3.3 cm	right site kidney laparoscopic nephrectomy	VHL mutation	was not observed
5.	37/female	ccRCC	3	pT1b	T1, N0, M0	6 cm	left site kidney resection	VHL mutation	was not observed
* 6.	72/female	ccRCC	2	pT1a	T1, N0, M0	2 × 1.5 cm	right site laparoscopic kidney nephrectomy	VHL mutation	cervical carcinoma
7.	36/female	AML	―	―	―	3.3 cm	right site kidney resection	VHL mutation	bladder cancer
8.	46/male	pRCC	2	pT1b	T1, N0, M0	5 cm	right site kidney resection, open surgery	PTEN mutation	was not observed

pT1a: tumor size is less than 4 cm and organ localized; pT1b: tumor size is more than 4 cm but less than 7 cm, and the tumor is organ localized; pT4: tumor is extended to adrenal gland, pRCC: papillary type of renal cell carcinoma; AML: angiomyolipoma. *: in this patient, 1.3 cm adenoma of adrenal glands and metastasis were observed on the axon sheet, and cervical carcinoma was developed as an associated tumor.

## Data Availability

The data presented in this study are available upon request to the corresponding author.

## References

[1] Bukowski R.M. (2009). Prognostic factors for survival in metastatic renal cell carcinoma: Update 2008. Cancer.

[2] Kumar A., Kumari N., Gupta V., Prasad R. (2018). Renal Cell Carcinoma: Molecular Aspects. Indian J. Clin. Biochem. IJCB.

[3] Schmidt L.S., Linehan W.M. (2016). Genetic predisposition to kidney cancer. Semin. Oncol..

[4] Wang J., Xi Z., Xi J., Zhang H., Li J., Xia Y., Yi Y. (2018). Somatic mutations in renal cell carcinomas from Chinese patients revealed by whole exome sequencing. Cancer Cell Int..

[5] Prasad S.R., Surabhi V.R., Menias C.O., Raut A.A., Chintapalli K.N. (2008). Benign renal neoplasms in adults: Cross-sectional imaging findings. AJR Am. J. Roentgenol..

[6] Younus M.S., Stephen W.L. Renal Angiomyolipoma. StatPearls. https://www.ncbi.nlm.nih.gov/books/NBK585104/.

[7] Muscarella L.A., D’Agruma L., la Torre A., Gigante M., Coco M., Parrella P., Battaglia M., Carrieri G., Carella M., Zelante L. (2013). VHL gene alterations in Italian patients with isolated renal cell carcinomas. Int. J. Biol. Markers.

[8] Dandanell M., Friis-Hansen L., Sunde L., Nielsen F.C., Hansen T.V. (2012). Identification of 3 novel VHL germ-line mutations in Danish VHL patients. BMC Med. Genet..

[9] Gao W., Li W., Xiao T., Liu X.S., Kaelin W.G. (2017). Inactivation of the PBRM1 tumor suppressor gene amplifies the HIF-response in VHL−/− clear cell renal carcinoma. Proc. Natl. Acad. Sci. USA.

[10] Kim H.S., Kim J.H., Jang H.J., Han B., Zang D.Y. (2018). Clinicopathologic Significance of VHL Gene Alteration in Clear-Cell Renal Cell Carcinoma: An Updated Meta-Analysis and Review. Int. J. Mol. Sci..

[11] Ge Y.Z., Xu L.W., Zhou C.C., Lu T.Z., Yao W.T., Wu R., Zhao Y.C., Xu X., Hu Z.K., Wang M. (2017). A BAP1 Mutation-specific MicroRNA Signature Predicts Clinical Outcomes in Clear Cell Renal Cell Carcinoma Patients with Wild-type BAP1. J. Cancer.

[12] Richard S., Gardie B., Couvé S., Gad S. (2013). Von Hippel-Lindau: How a rare disease illuminates cancer biology. Semin. Cancer Biol..

[13] Crespigio J., Berbel L.C.L., Dias M.A., Berbel R.F., Pereira S.S., Pignatelli D., Mazzuco T.L. (2018). Von Hippel-Lindau disease: A single gene, several hereditary tumors. J. Endocrinol. Investig..

[14] Kumar P.S., Venkatesh K., Srikanth L., Sarma P.V., Reddy A.R., Subramanian S., Phaneendra B.V. (2013). Novel three missense mutations observed in Von Hippel-Lindau gene in a patient reported with renal cell carcinoma. Indian J. Hum. Genet..

[15] Minardi D., Lucarini G., Milanese G., Montironi R., Di Primio R. (2017). Prognostic role of BAP1 in pT1 clear cell carcinoma in partial nephrectomy specimens. Virchows Arch..

[16] Testa J.R., Cheung M., Pei J., Below J.E., Tan Y., Sementino E., Cox N.J., Dogan A.U., Pass H.I., Trusa S. (2011). Germline BAP1 mutations predispose to malignant mesothelioma. Nat. Genet..

[17] Nasu M., Emi M., Pastorino S., Tanji M., Powers A., Luk H., Baumann F., Zhang Y.A., Gazdar A., Kanodia S. (2015). High Incidence of Somatic BAP1 alterations in sporadic malignant mesothelioma. J. Thorac. Oncol..

[18] Popova T., Hebert L., Jacquemin V., Gad S., Caux-Moncoutier V., Dubois-d’Enghien C., Richaudeau B., Renaudin X., Sellers J., Nicolas A. (2013). Germline BAP1 mutations predispose to renal cell carcinomas. Am. J. Hum. Genet..

[19] Sun C., Zhao C., Li S., Wang J., Zhou Q., Sun J., Ding Q., Liu M., Ding G. (2018). EZH2 Expression is increased in BAP1-mutant renal clear cell carcinoma and is related to poor prognosis. J. Cancer.

[20] Xu W., Yang Z., Zhou S.F., Lu N. (2014). Posttranslational regulation of phosphatase and tensin homolog (PTEN) and its functional impact on cancer behaviors. Drug Des. Dev. Ther..

[21] Dillon L.M., Miller T.W. (2014). Therapeutic targeting of cancers with loss of PTEN function. Curr. Drug Targets.

[22] Giudice F.S., Squarize C.H. (2013). The determinants of head and neck cancer: Unmasking the PI3K pathway mutations. J. Carcinog. Mutagen..

[23] Leslie N.R., Longy M. (2016). Inherited PTEN mutations and the prediction of phenotype. Semin. Cell Dev. Biol..

[24] Jin G., Kim M.J., Jeon H.S., Choi J.E., Kim D.S., Lee E.B., Cha S.I., Yoon G.S., Kim C.H., Jung T.H. (2010). PTEN mutations and relationship to EGFR, ERBB2, KRAS, and TP53 mutations in non-small cell lung cancers. Lung Cancer.

[25] Lynch E.D., Ostermeyer E.A., Lee M.K., Arena J.F., Ji H., Dann J., Swisshelm K., Suchard D., MacLeod P.M., Kvinnsland S. (1997). Inherited mutations in PTEN that are associated with breast cancer, cowden disease, and juvenile polyposis. Am. J. Hum. Genet..

[26] Marsh Durban V., Jansen M., Davies E.J., Morsink F.H., Offerhaus G.J., Clarke A.R. (2014). Epithelial-specific loss of PTEN results in colorectal juvenile polyp formation and invasive cancer. Am. J. Pathol..

[27] Minaguchi T., Yoshikawa H., Oda K., Ishino T., Yasugi T., Onda T., Nakagawa S., Matsumoto K., Kawana K., Taketani Y. (2001). PTEN mutation located only outside exons 5, 6, and 7 is an independent predictor of favorable survival in endometrial carcinomas. Clin. Cancer Res..

[28] Yang J., Ren Y., Wang L., Li B., Chen Y., Zhao W., Xu W., Li T., Dai F. (2010). PTEN mutation spectrum in breast cancers and breast hyperplasia. J. Cancer Res. Clin. Oncol..

[29] Aguissa-Touré A.H., Li G. (2012). Genetic alterations of PTEN in human melanoma. Cell Mol. Life Sci..

[30] Baig R.M., Mahjabeen I., Sabir M., Masood N., Hafeez S., Malik F.A., Kayani M.A. (2011). Genetic changes in the PTEN gene and their association with breast cancer in Pakistan. Asian Pac. J. Cancer Prev..

[31] Malentacchi F., Turrini I., Sorbi F., Projetto E., Castiglione F., Fambrini M., Petraglia F., Pillozzi S., Noci I. (2019). Pilot investigation of the mutation profile of PIK3CA/PTEN genes (PI3K pathway) in grade 3 endometrial cancer. Oncol. Rep..

[32] Wen Y.G., Wang Q., Zhou C.Z., Qiu G.Q., Peng Z.H., Tang H.M. (2010). Mutation analysis of tumor suppressor gene PTEN in patients with gastric carcinomas and its impact on PI3K/AKT pathway. Oncol. Rep..

[33] Yin L., Liu C.X., Nong W.X., Chen Y.Z., Qi Y., Li H.A., Hu W.H., Sun K., Li F. (2012). Mutational analysis of p53 and PTEN in soft tissue sarcoma. Mol. Med. Rep..

[34] Mattocks C., Tarpey P., Bobrow M., Whittaker J. (2000). Comparative sequence analysis (CSA): A new sequence-based method for the identification and characterization of mutations in DNA. Hum. Mutat..

[35] Yoshida M., Ashida S., Kondo K., Kobayashi K., Kanno H., Shinohara N., Shitara N., Kishida T., Kawakami S., Baba M. (2000). Germ-line mutation analysis in patients with von Hippel-Lindau disease in Japan: An extended study of 77 families. Jpn. J. Cancer Res..

[36] Chen F., Kishida T., Yao M., Hustad T., Glavac D., Dean M., Gnarra J.R., Orcutt M.L., Duh F.M., Glenn G. (1995). Germline mutations in the von Hippel-Lindau disease tumor suppressor gene: Correlations with phenotype. Hum. Mutat..

[37] Leonardi E., Martella M., Tosatto S.C., Murgia A. (2011). Identification and in silico analysis of novel von Hippel-Lindau (VHL) gene variants from a large population. Ann. Hum. Genet..

[38] Whaley J.M., Naglich J., Gelbert L., Hsia Y.E., Lamiell J.M., Green J.S., Collins D., Neumann H.P., Laidlaw J., Li F.P. (1994). Germ-line mutations in the von Hippel-Lindau tumor-suppressor gene are similar to somatic von Hippel-Lindau aberrations in sporadic renal cell carcinoma. Am. J. Hum. Genet..

[39] Lv C., Bai Z., Liu Z., Luo P., Zhang J. (2015). Renal cell carcinoma risk is associated with the interactions of APOE, VHL and MTHFR gene polymorphisms. Int. J. Clin. Exp. Pathol..

[40] Butler M.G., Dasouki M.J., Zhou X.P., Talebizadeh Z., Brown M., Takahashi T.N., Miles J.H., Wang C.H., Stratton R., Pilarski R. (2005). Subset of individuals with autism spectrum disorders and extreme macrocephaly associated with germline PTEN tumour suppressor gene mutations. J. Med. Genet..

[41] Zhang N., Wang X., Tang Z., Qiu X., Guo Z., Huang D., Xiong H., Guo Q. (2020). The Correlation Between Tuberous Sclerosis Complex Genotype and Renal Angiomyolipoma Phenotype. Front. Genet..

[42] Song X., Peng Y., Wang X., Chen Y., Jin L., Yang T., Qian M., Ni W., Tong X., Lan J. (2018). Incidence, Survival, and Risk Factors for Adults with Acute Myeloid Leukemia Not Otherwise Specified and Acute Myeloid Leukemia with Recurrent Genetic Abnormalities: Analysis of the Surveillance, Epidemiology, and End Results (SEER) Database, 2001–2013. Acta Haematol..

[43] Fiske J., Patel R., Kau E., Pappas J.G., Garcia R.A., Taneja S.S. (2005). Multifocal renal oncocytoma in a patient with Von Hippel-Lindau mutation. Urology.

[44] Eckel-Passow J.E., Serie D.J., Cheville J.C., Ho T.H., Kapur P., Brugarolas J., Thompson R.H., Leibovich B.C., Kwon E.D., Joseph R.W. (2017). BAP1 and PBRM1 in metastatic clear cell renal cell carcinoma: Tumor heterogeneity and concordance with paired primary tumor. BMC Urol..

[45] Farley M.N., Schmidt L.S., Mester J.L., Pena-Llopis S., Pavia-Jimenez A., Christie A., Vocke C.D., Ricketts C.J., Peterson J., Middelton L. (2013). A novel germline mutation in BAP1 predisposes to familial clear-cell renal cell carcinoma. Mol. Cancer Res..

[46] Brenner W., Färber G., Herget T., Lehr H.A., Hengstler J.G., Thüroff J.W. (2002). Loss of tumor suppressor protein PTEN during renal carcinogenesis. Int. J. Cancer.

[47] Wang S.S., Gu Y.F., Wolff N., Stefanius K., Christie A., Dey A., Hammer R.E., Xie X.J., Rakheja D., Pedrosa I. (2014). Bap1 is essential for kidney function and cooperates with Vhl in renal tumorigenesis. Proc. Natl. Acad. Sci. USA.

[48] Teh B.T., Blennow E., Giraud S., Sahlén S., Hii S.I., Brookwell R., Brauch H., Nordenskjöld M., Larsson C., Nicol D. (1998). Bilateral multiple renal oncocytomas and cysts associated with a constitutional translocation (8;9)(q24.1;q34.3) and a rare constitutional VHL missense substitution. Genes Chromosomes Cancer.

[49] Chrabańska M., Szweda-Gandor N., Drozdzowska B. (2023). Two Single Nucleotide Polymorphisms in the Von Hippel-Lindau Tumor Suppressor Gene in Patients with Clear Cell Renal Cell Carcinoma. Int. J. Mol. Sci..

[50] Webster B.R., Gopal N., Ball M.W. (2022). Tumorigenesis Mechanisms Found in Hereditary Renal Cell Carcinoma: A Review. Genes.

[51] Hussain A., Pandith A.A., Shah Z.A., Wani M.S. (2013). The Profound Impact of von Hippel-Lindau Gene Mutations in Renal Cell Cancers: A Study of the Kashmiri Population. UroToday Int. J..

